# Strain heterogeneity, cooccurrence network, taxonomic composition and functional profile of the healthy ocular surface microbiome

**DOI:** 10.1186/s40662-021-00228-4

**Published:** 2021-02-24

**Authors:** Yutong Kang, Shudan Lin, Xueli Ma, Yanlin Che, Yiju Chen, Tian Wan, Die Zhang, Jiao Shao, Jie Xu, Yi Xu, Yongliang Lou, Meiqin Zheng

**Affiliations:** 1 Eye Hospital and School of Ophthalmology and Optometry, Wenzhou Medical University, Wenzhou, Zhejiang China; 2 National Clinical Research Center for Ocular Diseases, Wenzhou, Zhejiang China; 3 Wenzhou Key Laboratory of Sanitary Microbiology, Key Laboratory of Laboratory Medicine, Ministry of Education, School of Laboratory Medicine and Life Sciences, Wenzhou Medical University, Wenzhou, Zhejiang 325035 China

**Keywords:** Healthy ocular surface microbiome, Cooccurrence network, Functional composition, Strain level

## Abstract

**Background:**

There is growing evidence indicating that the microbial communities that dwell on the human ocular surface are crucially important for ocular surface health and disease. Little is known about interspecies interactions, functional profiles, and strain heterogeneity across individuals in healthy ocular surface microbiomes.

**Methods:**

To comprehensively characterize the strain heterogeneity, cooccurrence network, taxonomic composition and functional profile of the healthy ocular surface microbiome, we performed shotgun metagenomics sequencing on ocular surface mucosal membrane swabs of 17 healthy volunteers.

**Results:**

The healthy ocular surface microbiome was classified into 12 phyla, 70 genera, and 140 species. The number of species in each healthy ocular surface microbiome ranged from 6 to 47, indicating differences in microbial diversity among individuals. The species with high relative abundances and high positivity rates were *Streptococcus pyogenes*, *Staphylococcus epidermidis*, *Propionibacterium acnes*, *Corynebacterium accolens*, and *Enhydrobacter aerosaccus*. A correlation network analysis revealed a competitive interaction of *Staphylococcus epidermidis* with *Streptococcus pyogenes* in ocular surface microbial ecosystems. *Staphylococcus epidermidis* and *Streptococcus pyogenes* revealed phylogenetic diversity among different individuals. At the functional level, the pathways related to transcription were the most abundant. We also found that there were abundant lipid and amino acid metabolism pathways in the healthy ocular surface microbiome.

**Conclusion:**

This study explored the strain heterogeneity, cooccurrence network, taxonomic composition, and functional profile of the healthy ocular surface microbiome. These findings have important significance for the future development of probiotic-based eye therapeutic drugs.

**Supplementary Information:**

The online version contains supplementary material available at 10.1186/s40662-021-00228-4.

## Background

Human mucosal surfaces are normally colonized by diverse microbial flora [1]. As the most exposed mucosal tissue of the human body, the ocular surface is susceptible to insult by environmental factors, such as microorganisms [2]. Although a healthy ocular surface can live in harmony with symbiotic microorganisms, microbial community imbalance or transient flora increases on the ocular surface may lead to diseases [3]. Bacteria are considered major contributors to ocular infections worldwide. Ocular infections, if not promptly treated, can cause vision impairment and blindness [4]. In the Human Microbiome Project, much effort has been applied to characterize the human mucosal microbiome, spanning the gut, mouth, respiratory tract, skin, and urogenital tract [5]. However, in the field of visual research, this aspect is still in its infancy.

Previous culture-based surveys indicated that the microbial flora colonizing the ocular surface is dominated by gram-positive *Firmicutes* [6]. Whereas conventional methods have difficulty detecting microorganisms that are rarely encountered, grow slowly, and cannot be cultured, next-generation sequencing (NGS) technologies have provided a much more detailed picture of the healthy ocular surface microbiome [7–11]. Although the precise distribution of each phylum is different between individuals, the most consistent contributors on the ocular surface are *Proteobacteria*, *Firmicutes*, and *Actinobacteria* [3, 7, 10, 12, 13].

A number of studies in the intestine and other microbiome sites have shown that cohabiting microorganisms within a microbiome maintain a stable state of competition and cooperation, such as competing with one another for or mutually utilizing resources, nutrition, and space [14, 15]. The abundance of each member of the microbiome is constrained by the optimal ratio to maintain homeostasis [14]. Interactions among microbial species are crucial for the sustainability of various ecosystems [16]. However, our current understanding of many interaction relationships among the components of the ocular surface microecosystem is still very limited.

The microbial communities that dwell on the human ocular surface are important for ocular surface health and disease [17–22]. The microbial flora populating the human ocular surface has been characterized with NGS, but few studies have focused on strain-level resolution. Although high-quality studies have been conducted on the genetic variation and population structure of humans and how population heritability is shaped, the variation and structure of bacterial cells residing in the human body are relatively unknown [23–25]. Strain-level analysis of the gut microbiome has shown that in common species, different strains of species are associated with different individuals [26].

Most previous studies used mainly 16S rRNA gene sequencing, which did not allow for confident classification of the microbiota to the level of (sub)species [27], and 16S rRNA gene sequencing cannot provide data regarding functional genes and can indicate only the potential functionality of microbial communities. Our metagenome sequencing data provide more direct functional information and higher taxonomic resolution for a comprehensive understanding of the taxonomic and functional compilations of the healthy ocular surface microbiome. Species interactions and strain heterogeneity among individuals have received little attention in past studies based on shotgun metagenomics sequencing for the characterization of normal ocular surface microecology [28, 29]. Here, we demonstrate the individual differences in *Staphylococcus epidermidis* and *Streptococcus pyogenes* at the strain level. We also found the existence of a competing relationship between *Staphylococcus epidermidis* and *Streptococcus pyogenes* in the cooccurrence network.

## Methods

### Ethics approval and consent to participate

This study was approved by the Ethics Committee of the Eye Hospital of Wenzhou Medical University (number: KYK [2017] 23) and adhered to the tenets of the Declaration of Helsinki. All subjects provided written informed consent at the time of sample collection.

### Sample collection and processing

A total of 8 male subjects (age 41.6 ± 13.7 years) and 9 female subjects (age 43 ± 13.3 years) with healthy ocular surfaces were recruited from communities across Zhejiang, China. All subjects received systematic eye examinations by the same ophthalmologist before sample collection. In addition, all subjects were requested to fill in an ocular surface disease index (OSDI) screening questionnaire to evaluate ocular discomfort. The overall OSDI screening questionnaire scores defined the ocular surface as normal (0–12 points) [30]. The overall OSDI screening questionnaire scores of all subjects in this study were 5 or less. The exclusion criteria of the study were as follows: (i) history of systemic or (ii) ocular diseases or (iii) contact lens wearing and (iv) topical or systemic antibiotics, steroid, any eye drop (prescribed or over the counter) or probiotic treatment within 6 months. The subject’s eye was administered sterile topical proparacaine. After topical anesthesia for 1 to 3 min, the subject looked upward for sample collection. Samples were taken from the ocular surface mucosal tissues (upper and lower palpebral, caruncle, and conjunctival fornix) using flocked swabs and stored in a Copan ESwab transport system (Copan Diagnostics Inc., Murrieta, CA) on ice blocks. Upon return to the laboratory, the swabs were frozen at − 80 °C until further processing. Genomic DNA was extracted from the swabs using pathogen lysis tubes L (QIAGEN, Hilden, Germany) and a QIAamp UCP Pathogen Mini Kit (QIAGEN, Hilden, Germany) according to the manufacturer’s instructions. The DNA concentration was measured using a Qubit® 2.0 Fluorometer.

### Prevention of contamination and negative controls

To avoid contamination during sample collection, sample collections were carried out in an ophthalmic treatment room sterilized by ultraviolet light. Unused clean swabs were exposed to the sampling environment and waved in air for 10 s to collect field controls. Extraction controls were generated during DNA extraction to monitor for reagent contamination. Unused sterile flocked swabs moistened with sterile topical proparacaine were processed as anesthetic controls for DNA extraction. No DNA was detected in the field controls, extraction controls, or anesthetic controls using a Qubit® dsDNA Assay Kit and a Qubit® 2.0 Fluorometer.

We amplified the variable 4 (V4) region of the 16S ribosomal RNA gene extracted from the field controls, extraction controls, anesthetic controls, and actual samples using the universal primers 515F and 806R. PCR reagents without template DNA were used for PCR amplification as a negative control. The amplification system was 20 μl, consisting of 4 μl 5*FastPfu buffer, 2 μl 2.5 mM dNTPs, 0.8 μl primers (5 μM), 0.4 μl FastPfu polymerase, and 10 ng DNA template. Reaction mixtures were incubated for predenaturation at 95 °C for 3 min, 27 thermal cycles (denaturation at 95 °C for 30 s, annealing at 55 °C for 30 s, and extension at 72 °C for 30 s), and extension at 72 °C for 10 min (PCR instrument: ABI GeneAmp® 9700). Amplification products of the 16S rRNA gene V4 DNA region were visualized by agarose gel electrophoresis. Bright bands were observed at ∼290 bp in actual samples; no band was found in field controls, extraction controls, anesthetic controls, or PCR negative controls. Sequencing was performed on field controls, extraction controls, anesthetic controls, and PCR negative controls and did not yield any reads.

### Shotgun metagenomics sequencing

The above samples were used for shotgun metagenomics sequencing. Paired-end sequencing (150 bp × 2) was performed on the HiSeq X10 platform (Novogene Co., Ltd., Beijing, China). The quality of raw reads was assessed using FastQC software according to a previously described in-house bioinformatics pipeline [31]. After the adapter sequences were trimmed by the Cutadapt tool (http://code.google.com/p/cutadapt/), low-quality reads were removed using Trim Galore [32], and the remaining high-quality reads were visualized using SplicingViewer [33]. Bowtie2 was used to map trimmed reads to the human reference genome (hg19) to generate BAM files [34]. Aligned reads were removed using SAMtools to obtain clean nonhuman sequences [35]. MetaPhlAn2 was executed to generate taxonomic profiles, with default parameters [36]. Strain-level profiling was performed with StrainPhlAn [37]. The remaining metagenomic sequences for each sample were assembled with Megahit [38]. The contigs were submitted to Prokka for gene prediction [39]. Quantification of predicted genes was performed with Salmon [40]. Subsequently, redundant amino acid sequences were removed by using CD-HIT with a sequence identity threshold of 90% [41]. Functional annotations and transcription factor prediction were implemented using eggNOG-mapper [42], CollecTF [43], and ArchaeaTF [44]. Figures were visualized with R (version 3.6.2). The R vegan package was used to calculate the Shannon diversity index, inverse Simpson index, Bray-Curtis dissimilarity, and Jaccard index at the species level. Pairwise comparisons of alpha diversity indices were performed using the Wilcoxon rank-sum test. Permutational multivariate analysis of variance (PERMANOVA) of Bray-Curtis distances and Jaccard distances were performed for statistical analysis of beta diversity. Principal coordinate analysis (PCoA) was used to visualize the resulting distance matrix.

## Results

### Taxonomic composition

Illumina sequencing of all samples produced 1.35 billion reads. After filtering out reads matching the human genome sequence, an average of approximately 2.18 million microbial reads from each sample were obtained for further analysis. The healthy ocular surface microbiome was classified into 12 phyla, 20 classes, 29 orders, 50 families, 70 genera, and 140 species among all subjects (see Supplemental Figure 1). At the phylum level, 7 phyla had an average relative abundance > 1%, namely, *Actinobacteria, Bacteroidetes, Deinococcus-Thermus, Firmicutes, Proteobacteria, Eukaryota-noname,* and *Viruses-noname* (see Supplemental Figure 2a). The top three phyla (*Firmicutes* (average: 45.02%), *Actinobacteria* (28.45%), and *Proteobacteria* (16.17%) accounted for the majority. Interestingly, the healthy ocular surface microbiome in some samples was dominated by a single phylum. For instance, in the ocular microbiota of CON3 and CON10, *Firmicutes* accounted for 80.76 and 83.89%, respectively.

At the genus level, the 15 genera with more than 1% average relative abundance were *Streptococcus*, *Corynebacterium*, *Propionibacterium*, *Staphylococcus*, *Neisseria*, *Morganella*, *Escherichia*, *Shigella*, *Siphoviridae*, *Acinetobacter*, *Finegoldia*, *Anelloviridae*, *Alphatorquevirus*, *Brevundimonas*, and *Enhydrobacter* (see Supplemental Figure 2b). *Streptococcus* (average: 24.62%), *Staphylococcus* (14.15%), *Propionibacterium* (12.93%), and *Corynebacterium* (9.05%) were the top four genera. Similarly, the subjects CON10 and CON3 showed extreme dominance by *Streptococcus*, accounting for 83.89 and 79.49%, respectively.

**Fig. 1:**
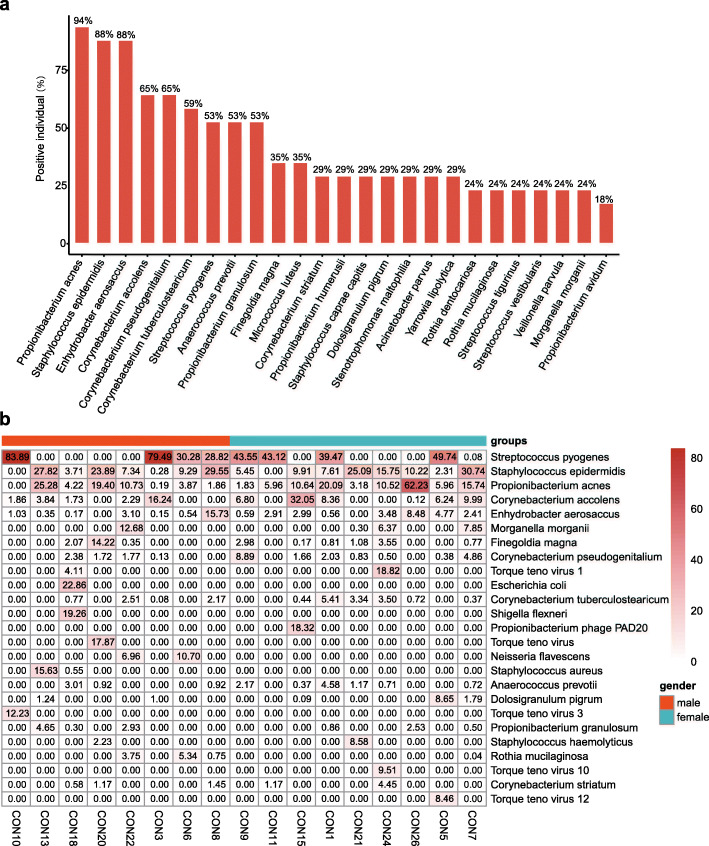
The positive rate and relative abundance of microbial species in the healthy ocular surface. **a** Species with a positive rate greater than 10% are shown. **b** The heat map shows the relative abundance of the top 25 species in each sample

At the species level, 19.59 ± 11.34 (range, 6–47) species were detected in the healthy ocular surface microbiome samples from each subject. No species was detected simultaneously in any subject (see Supplemental Figure 3). The taxonomic composition was unique to each sample. Among all species, *Propionibacterium acnes*, *Staphylococcus epidermidis*, *Enhydrobacter aerosaccus*, *Corynebacterium accolens*, *Corynebacterium pseudogenitalium*, *Corynebacterium tuberculostearicum*, *Streptococcus pyogenes*, *Anaerococcus prevotii*, and *Finegoldia magna* were detected in > 50% of healthy volunteers (Fig. 1a). Figure 1b shows the distribution and relative abundance of the top 25 species. The five most abundant species were *Streptococcus pyogenes*, *Staphylococcus epidermidis*, *Propionibacterium acnes*, *Corynebacterium accolens*, and *Enhydrobacter aerosaccus*.

**Fig. 2:**
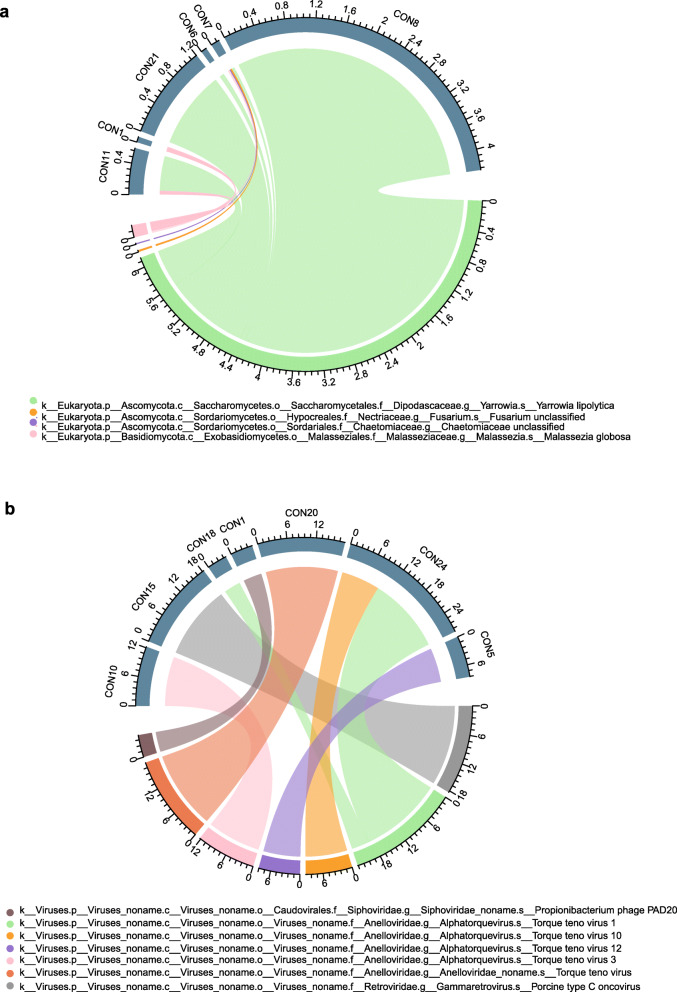
Chord diagram showing the distribution and relative abundance of (**a**) fungi and (**b**) viruses in the subjects

Bacteria were shared by all subjects, yet fungi and viruses were not found in all healthy ocular surface microbiomes. The positive rate for fungi was 35%, and that for viruses was 41%. The average relative abundances of bacteria, fungi and viruses were 93, 2, and 5%, respectively. The fungal microbiome was classified into 2 phyla (*Ascomycota* and *Basidiomycota*) and 4 genera (*Yarrowia*, *Chaetomiaceae, Fusarium,* and *Malassezia*) (Fig. 2a). Seven viruses, namely, *Propionibacterium phage PAD20*, *Torque teno virus 1*, *Torque teno virus 10*, *Torque teno virus 12*, *Torque teno virus 3*, *Torque teno virus*, and *Porcine type C oncovirus*, were detected (Fig. 2b).

### Strain heterogeneity

**Fig. 3:**
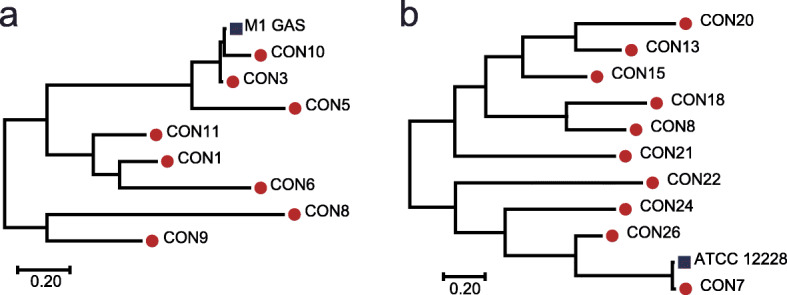
Maximum-likelihood trees derived from strain variation in individual bacteria inferred using StrainPhlAn. **a** *Streptococcus pyogenes*; **b** *Staphylococcus epidermidis*

StrainPhlAn allowed us to study the strain-level features of the healthy ocular surface microbiome, which targets single-nucleotide polymorphisms (SNPs) within clade-specific markers of strains in metagenomes. Among all species, *Streptococcus pyogenes* and *Staphylococcus epidermidis* with sufficient coverage were profiled by using the StrainPhlAn method. We constructed a phylogenetic tree of *Streptococcus pyogenes* (Fig. 3a) covering eight individuals and *Staphylococcus epidermidis* (Fig. 3b) covering 10 individuals from the metagenomic sequence data. Using SNP-based analysis, considerable strain-level heterogeneity was observed with respect to reference genomes (*Streptococcus pyogenes M1 GAS* and *Staphylococcus epidermidis ATCC 12228*). The common species from different individuals formed almost distinct branches, which could indicate the presence of different strains.

For *Streptococcus pyogenes* and *Staphylococcus epidermidis*, the strains were separated into two clusters, but neither of the clusters was age- or sex-specific. *Staphylococcus epidermidis* residing within subject CON7 was closely related to *Staphylococcus epidermidis* ATCC 12228. *Staphylococcus epidermidis* strains residing within CON21 and CON22 formed a monophyletic clade. *Streptococcus pyogenes M1 GAS* clustered together with *Streptococcus pyogenes* strains residing within CON3 and CON10, which indicated an intimate intraspecies relationship among them. In addition, *Streptococcus pyogenes* residing within CON3 (*Streptococcus pyogenes CON3*) branched near the base of *Streptococcus pyogenes M1 GAS* and *Streptococcus pyogenes* residing within CON10 (*Streptococcus pyogenes CON10*). We inferred that *Streptococcus pyogenes CON3* likely arose before *Streptococcus pyogenes M1 GAS* and *Streptococcus pyogenes CON10*.

### Interaction network

**Fig. 4:**
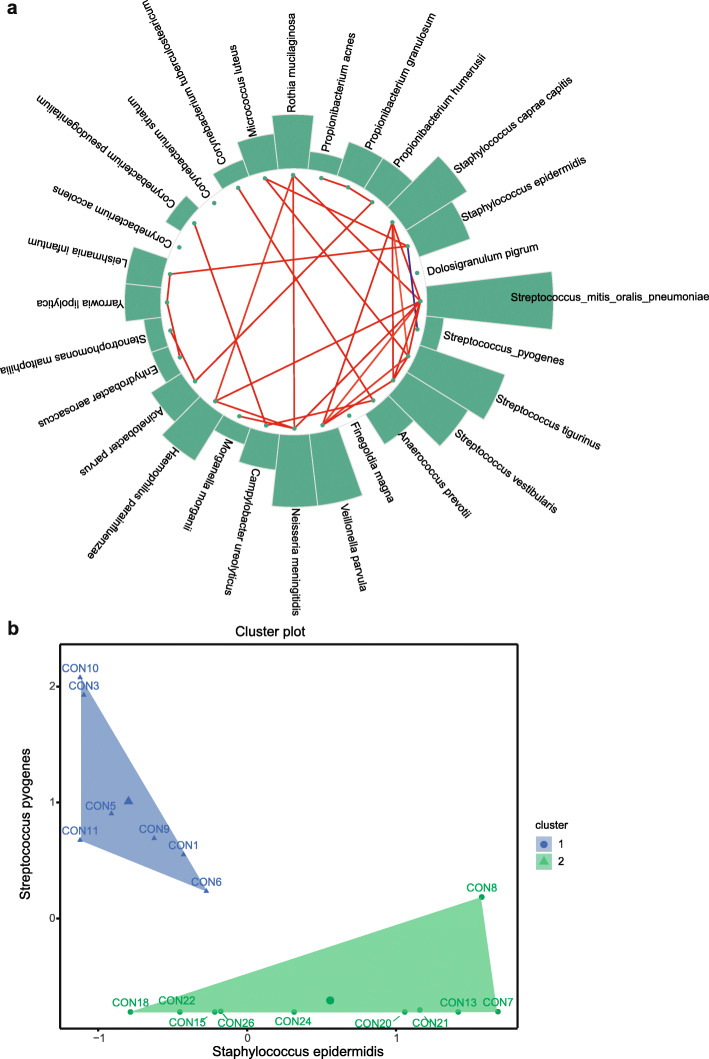
Microbial cooccurrence in the healthy ocular surface microbiome and K-means clustering for unsupervised grouping. **a** Interaction network of species with an average relative abundance greater than 0.1% and appearing in more than two subjects. The colored lines represent correlations between species; red is a positive correlation, and blue is a negative correlation. The heights of the bars are positively correlated with the species’ degrees of connectivity. **b** K-means clustering (k = 2) performed with the relative abundances of *Staphylococcus epidermidis* and *Streptococcus pyogenes*

An interaction network was constructed for species with a mean relative abundance greater than 0.1% that appeared in more than two subjects (Fig. 4a). *Streptococcus_mitis_oralis_pneumoniae* had the most interactions, suggesting key roles in the network. *Streptococcus_mitis_oralis_pneumoniae* exhibited only positive interactions with 7 species, *Staphylococcus_caprae_capitis*, *Rothia mucilaginosa*, *Haemophilus parainfluenzae*, *Neisseria meningitidis*, *Veillonella parvula*, *Streptococcus vestibularis*, and *Streptococcus tigurinus*. Interestingly, only *Staphylococcus epidermidis* was negatively related to *Streptococcus pyogenes*, implying that there may be competitive inhibition between them. Based on this result, subjects were clustered by the k-means algorithm with k = 2, using the relative abundance of *Streptococcus pyogenes* and *Staphylococcus epidermidis*. Unsupervised clustering generated two distinct clusters: cluster 1 and cluster 2 (Fig. 4b). In cluster 1, the relative abundance of *Streptococcus pyogenes* in each sample was > 30%.

### The effects of age and sex on the composition of healthy ocular surface microbiomes

The subjects were grouped by sex (female versus male) and age (≤40 versus > 40 years old) to investigate the influence of age and sex on the normal ocular surface microbiome. Alpha diversity analysis based on the Shannon and inverse Simpson diversity indices revealed that there was no significant variation between sexes (see Supplemental Figure 4a, b) and ages (see Supplemental Figure 5a, b). PCoA based on the Bray-Curtis dissimilarity Jaccard index showed no effect on bacterial community structure for sex (see Supplemental Figure 4c, d) or age (see Supplemental Figure 5c, d).

### Functional pathways

**Fig. 5:**
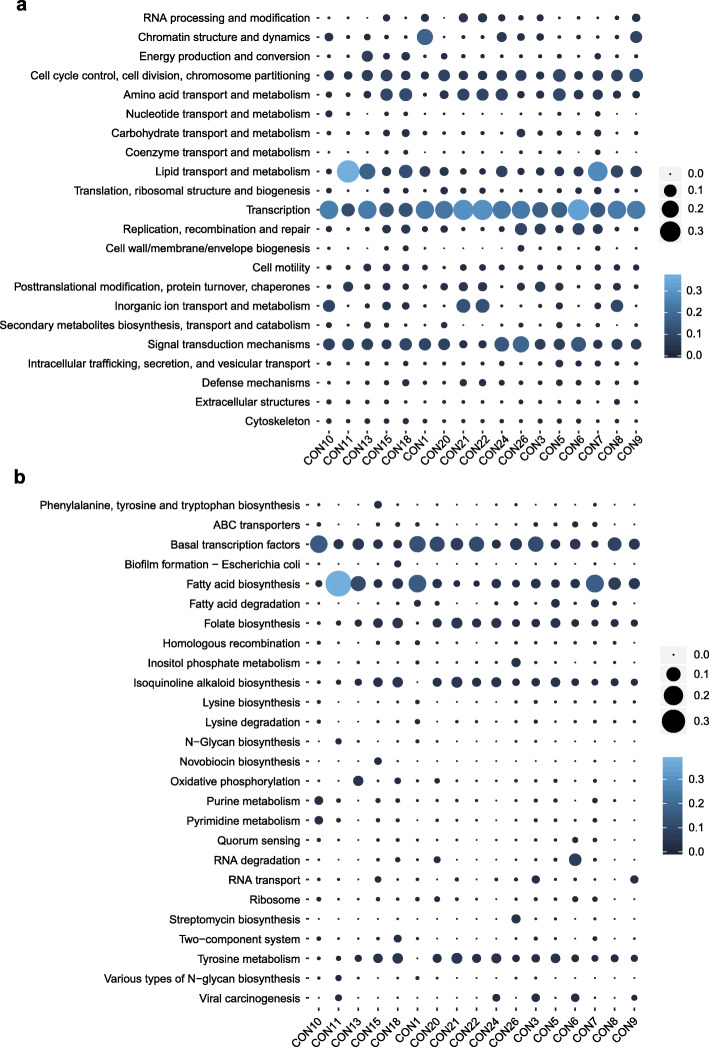
COG clusters (**a**) and KEGG (**b**) pathway heatmap

At present, the functional compositions of the healthy ocular surface microbiome are still poorly understood. Clusters of Orthologous Groups (COG) analysis revealed a total of 22 categories (Fig. 5a). Among these, 6 COG functional features were associated with metabolism, namely, amino acid transport and metabolism, nucleotide transport and metabolism, carbohydrate transport and metabolism, coenzyme transport and metabolism, lipid transport and metabolism, and inorganic ion transport and metabolism. Transcription was the most abundant COG annotation, followed by lipid transport and metabolism, signal transduction mechanisms, cell cycle control, cell division, chromosome partitioning and amino acid transport and metabolism. Figure 5b shows the microbial Kyoto Encyclopedia of Genes and Genomes (KEGG) pathways with a mean relative abundance greater than 0.1%. Similar to the COG annotation results, fatty acid biosynthesis accounted for the largest proportion, followed by basal transcription factors, folate biosynthesis, tyrosine metabolism, and isoquinoline alkaloid biosynthesis. Of note, *Escherichia coli* biofilm formation and viral carcinogenesis might be potential pathogenic pathways.

## Discussion

The microbial community populated on the human ocular surface may play an important role in both innate and adaptive immune responses. Although these normal commensal florae can colonize the healthy ocular surface without causing disease, several studies have revealed that alterations in the healthy ocular surface microbiome are associated with some ocular surface diseases, such as trachoma [12], keratitis [13, 45, 46], conjunctivitis [47], dry eye [48], mesangial gland dysfunction [49, 50], chronic Stevens-Johnson syndrome [51], and blepharitis [52]. Hence, a comprehensive understanding of the composition of the ocular surface microbiome is crucial for the future development of probiotic-based eye treatment drugs. Whereas existing research on the healthy ocular surface microbiome has used mainly 16S rRNA gene sequencing, in this study, we performed shotgun metagenomics sequencing on the ocular surface mucosal membrane swabs of 17 healthy volunteers and clearly described the taxonomic composition, the interspecies interactions, strain-level heterogeneity among different individuals, and functional profiles of the healthy ocular surface microbiome.

At the phylum level, the ocular surface microbial community was dominated by *Proteobacteria*, *Firmicutes* and *Actinomycetes*, which is consistent with previous studies [3, 7, 10, 12, 13]. Only two fungal phyla were found, namely, *Ascomycota* and *Basidiomycota*. Compared with a study based on internal transcribed spacer (ITS) sequencing to characterize the ocular surface fungal microbiome [45, 53, 54], we found fewer fungal species; this difference may be due to different sequencing methods. In shotgun metagenomics sequencing, the genomes of eukaryotes are usually very long and composed of many noncoding regions, which leads to poor read utilization [55–57]. For viruses, 5 different strains of *Torque teno virus* were found on the ocular surface of 5 subjects. *Torque teno virus* has been identified in culture-negative endophthalmitis [58], but the mechanism of how it causes endophthalmitis remains unclear.

Our results show that age and sex have no effect on the composition of the healthy ocular surface microbiome. There is still controversy about whether sex affects the composition of the ocular surface microbial community, and previous studies have shown mixed results [7, 12, 29, 59]. Future studies should expand the age and sex coverage of subjects and measure their sex hormones, such as estrogen, progesterone, androgens, prolactin, follicle-stimulating hormone, luteinizing hormone, testosterone, progesterone, and estradiol. Exploring the relationship between the level of sex hormones in the body and the diversity of the ocular surface microbial community will better reveal the influence of different sexes on the ocular surface microbiome.

*Staphylococcus epidermidis* is representative of the normal ocular surface flora and is the species most often isolated from the human eye surface. A previous study on the healthy ocular surface using shotgun metagenomics sequencing reported a high positivity rate for *Staphylococcus epidermidis* (73%) in surveyed subjects [29]. Our data showed a *Staphylococcus epidermidis* positive rate of 88%. Among these, 10 samples contained enough *Staphylococcus epidermidis* reads for strain assignment. Phylogenetic tree analysis showed that variation was observed across clades and individual genomes. Earlier studies based on pulsed-field gel electrophoresis also found polyclonality of *Staphylococcus epidermidis* on the healthy ocular surface [60]. Therefore, it is necessary to perform pangenome sequencing on *Staphylococcus epidermidis* strains isolated from the healthy ocular surface in the future to evaluate the difference in the function and virulence of different strains.

Interestingly, the healthy ocular surface microbiome of some subjects was dominated by *Streptococcus pyogenes*, which is a common bacterium isolated from infected eyes. Thus, the healthy ocular surface can achieve homeostasis with causative pathogens. Pathogens that cause eye infections may be introduced from the external environment, and when homeostasis of the eye is disrupted, commensal potential pathogens escape control and become pathogenic [61, 62]. Whether individuals carrying a high proportion of potential pathogens on the ocular surface are more likely to develop infections requires further research. Phylogenetic analysis showed that there were also individual differences among *Streptococcus pyogenes* at the strain level, which might be due to host age, living environment, and previous drug use.

A previous study investigated the stability of the composition of the ocular surface microbial community over time [7]. Some genera were present in subjects at all time points (baseline, 1 month, 3 months), which indicated that some taxa have longitudinal stability at the individual level. There is a necessity and is of much interest to study the degree of variability of the strain genomes within an individual over time. Future research should sample the same subject several times in a shorter or longer time interval to reveal the variability degree of the same-strain within-subject changes over time.

Another noteworthy finding is the functional components of the healthy ocular surface microbiome. Both COG and KEGG pathway analyses revealed high abundance of lipid metabolism (biosynthesis, degradation, and transport) pathways. This result may suggest that the healthy ocular surface microbiome plays an important role in lipid metabolism in the eye. The lipid layer can enhance tear distribution, maintain tear stability and prevent evaporation [63]. Recently, lipid formulations have been promoted to regulate dry eye and have achieved good results [64, 65]. Commercially available lipid formulations, including emulsions and liposomes, take the form of eye drops or sprays [66]. Our research provides important ideas for the future development of probiotic-based dry eye treatment. In future research, we will perform metagenomic sequencing on the healthy ocular surface microbiome of patients with dry eye to investigate whether the microbial lipid metabolism is reduced.

In addition to lipids, COG annotation results showed that there are also abundant amino acid metabolic pathways on the ocular surface. It has been reported that amino acids could be abundantly produced by *Corynebacterium* spp. [67]. In our analysis, several *Corynebacterium* spp. were detected in more than 50% of the healthy volunteers (Fig. 1a), indicating that *Corynebacterium* may play a potential role in these amino acid metabolic pathways. The amino acid metabolism pathway in the KEGG annotation results that had the highest proportion was tyrosine metabolism. Amino acids naturally exist in human tears and play a positive role in maintaining ocular surface homeostasis. A series of published clinical data indicate that the use of topical eye drops with amino acids supplemented can be beneficial to the healing of ocular surface diseases [68]. Whether the healthy ocular surface microbiome is involved in the occurrence and role of amino acids in tears is still unknown; a direction worth exploring in future studies.

We also found elevated frequencies of COG annotations related to inorganic ion transport and metabolism. Sphingomyelin are one of the major classes of anionic lipids in human tears [69]. Divalent cations may interact with the phosphate head groups of these phospholipids to help stabilize the lipids in tears [70]. The increase in divalent cations in tear fluid may change the folding of proteins [71, 72] and the interaction between proteins, which in turn affect the stability and surface tension of the tear film [73]. Exploring whether the ocular surface microbiome affects the stability of the tear film through the transport and metabolism of inorganic ions has important guiding value for the future development of novel methods that can increase tear film stability and eye comfort.

Probiotics are defined by the World Health Organization (WHO) as “live microorganisms that confer demonstrated health benefits for the host when ingested or topically applied”. Some research has been devoted to utilizing probiotics as topical ocular products [74, 75]. The ocular surface is a wide mucosal surface exposed to the external environment, which contributes largely to the type and number of microorganisms colonizing its surface. As the environment changes, the colonizing organisms may change accordingly. One possibility is that the ocular microbiome is formed by two populations: one stable, tightly embedded population in the ocular surface that is less susceptible to changes and a variable, more superficial population that is sensitive to recent environmental changes, which could be washed away by an ocular rinse. It is necessary to study whether after a thorough ocular rinse, a more stable microbiome is found in consecutive analyses, which could be relevant for ocular health. Furthermore, this information could suggest what the ‘good’ microorganisms are to be used in a topical probiotic formulation.

The limitations of this study are as follows. First, the sample size and geographical representation are limited, and thus limits the generalizability of our results to the entire population. Second, the use of anesthetic eye drops before sampling could lead to reduced diversity of ocular surface microbial communities.

## Conclusions

In summary, the healthy ocular surface microbiome was clearly demonstrated by the shotgun metagenomics survey in this study. The present study provides new directions for further studies on the healthy ocular surface microbiome.

## Supplementary Information

**Additional file 1: Supplemental Figure 1**. The number of different classification levels in all samples.

**Additional file 2: Supplemental Figure 2.** The major taxa with a mean relative abundance greater than 1% are presented. (a) Major phyla; (b) major genera. “Other” represents groupings of less abundant taxa (<1%).

**Additional file 3: Supplemental Figure 3.** Petal map based on the number of species. The center is the number of species shared by all samples, and the number on the petal shows the number of species specific to each sample.

**Additional file 4: Supplemental Figure 4.** Alpha and beta diversity of healthy ocular surface microbiota in male and female subjects. Shannon (a) and inverse Simpson indices (b) were used to estimate the level of diversity of the microbiota of the male and female groups. PCoA plots of Bray-Curtis (c) and Jaccard (d) distance matrices between the male and female groups.

**Additional file 5: Supplemental Figure 5.** Alpha and beta diversity of healthy ocular surface microbiota in young and old subjects. Shannon (a) and inverse Simpson indices (b) were used to estimate the level of diversity of the microbiota of the young and old groups. PCoA plots of Bray-Curtis (c) and Jaccard (d) distance matrices between the male and female groups.

## Data Availability

The datasets used and/or analyzed in this study are available from the corresponding author upon reasonable request.

## Abbreviations

NGS: Next-generation sequencing
COG: Clusters of Orthologous Groups
KEGG: Kyoto Encyclopedia of Genes and Genomes
SNPs: Single-nucleotide polymorphisms
PCoA: Principal coordinate analysis
ITS: Internal transcribed spacer
WHO: World Health Organization
PERMANOVA: permutational multivariate analysis of variance
*Streptococcus pyogenes CON3*: *Streptococcus pyogenes* residing within CON3
*Streptococcus pyogenes CON10*: *Streptococcus pyogenes* residing within CON10
