# Supplementary figures and images for: Strain heterogeneity, cooccurrence network, taxonomic composition and functional profile of the healthy ocular surface microbiome
Source: Eye Vis (Lond). 2021 Feb 24;8:6. doi: 10.1186/s40662-021-00228-4 (PMC7903678; doi:10.1186/s40662-021-00228-4)

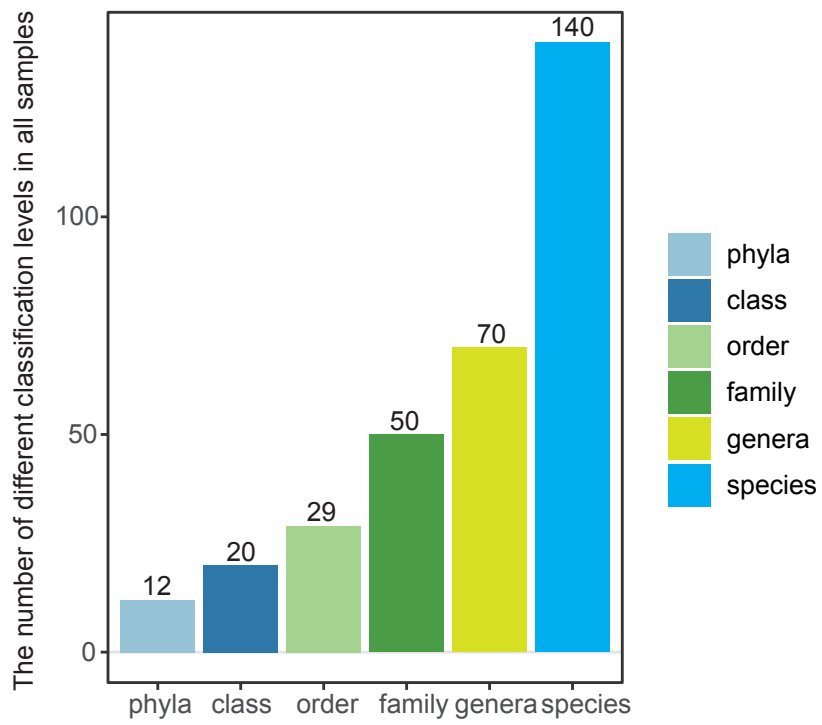

Supplement: Supplementary file 1 — Additional file 1: Supplemental Figure 1. The number of different classification levels in all samples. [file 40662_2021_228_MOESM1_ESM.pdf]

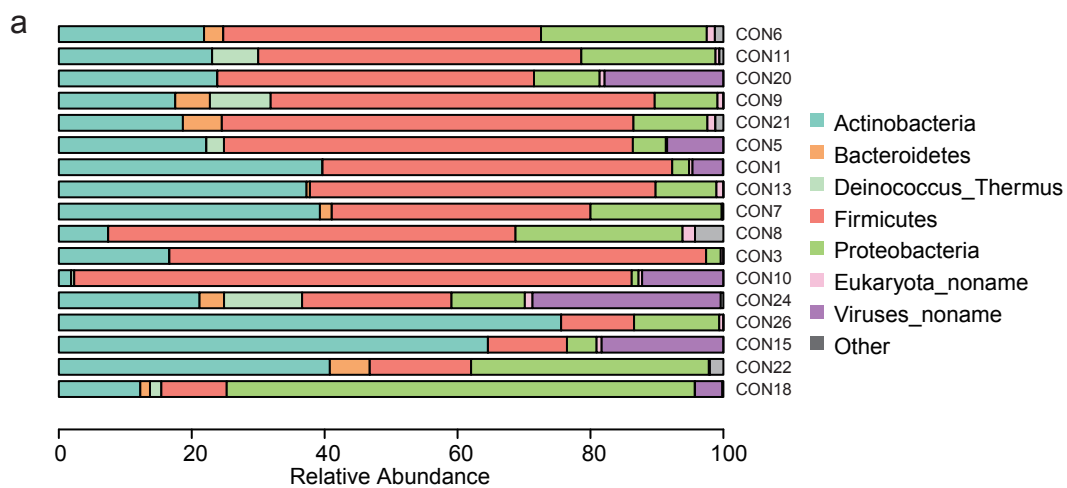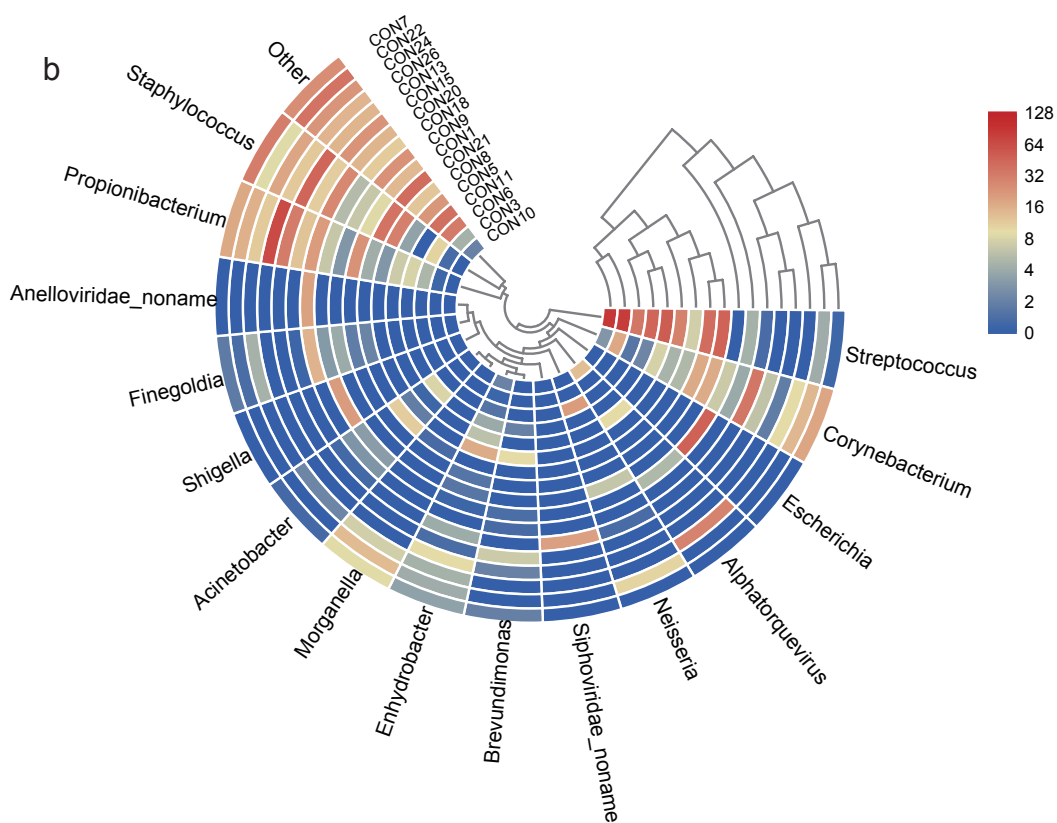

Supplement: Supplementary file 2 — Additional file 2: Supplemental Figure 2. The major taxa with a mean relative abundance greater than 1% are presented. (a) Major phyla; (b) major genera. “Other” represents groupings of less abundant taxa (<1%). [file 40662_2021_228_MOESM2_ESM.pdf]

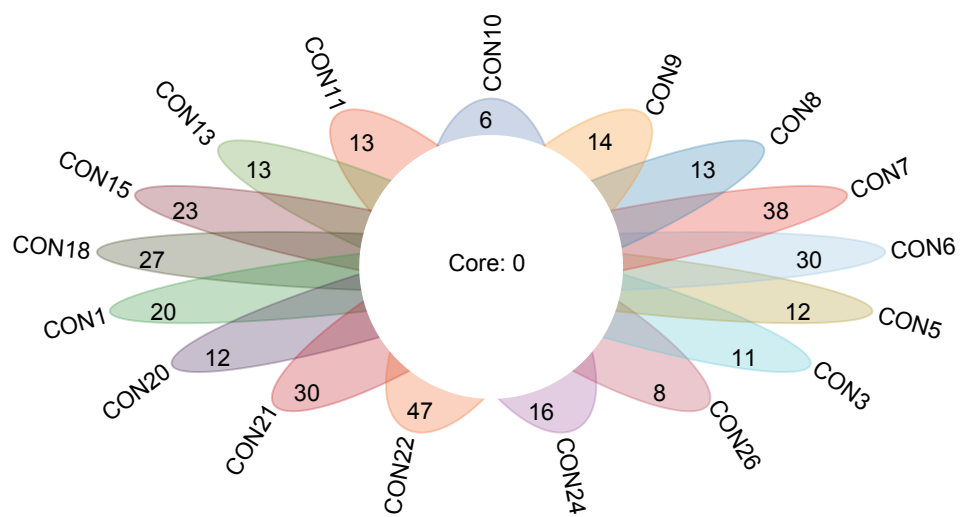

Supplement: Supplementary file 3 — Additional file 3: Supplemental Figure 3. Petal map based on the number of species. The center is the number of species shared by all samples, and the number on the petal shows the number of species specific to each sample. [file 40662_2021_228_MOESM3_ESM.pdf]

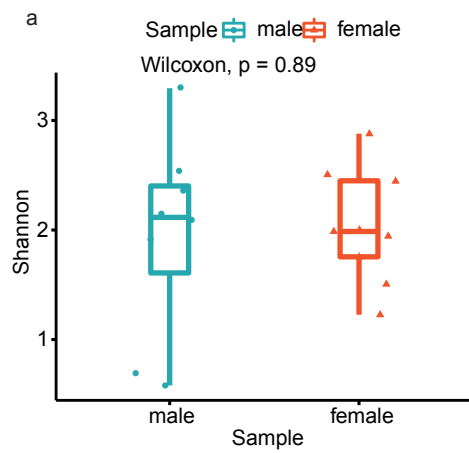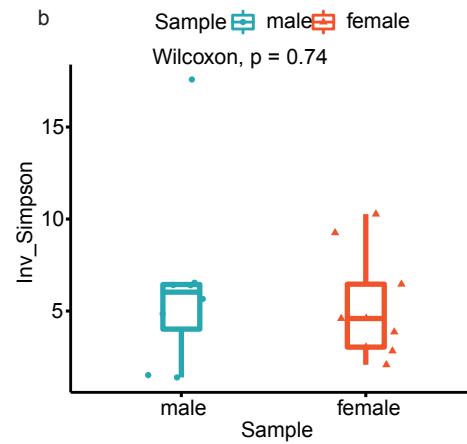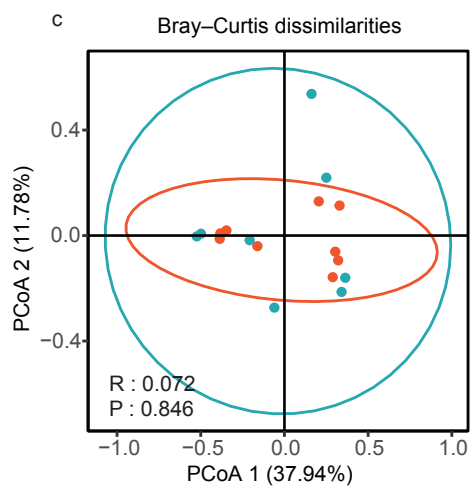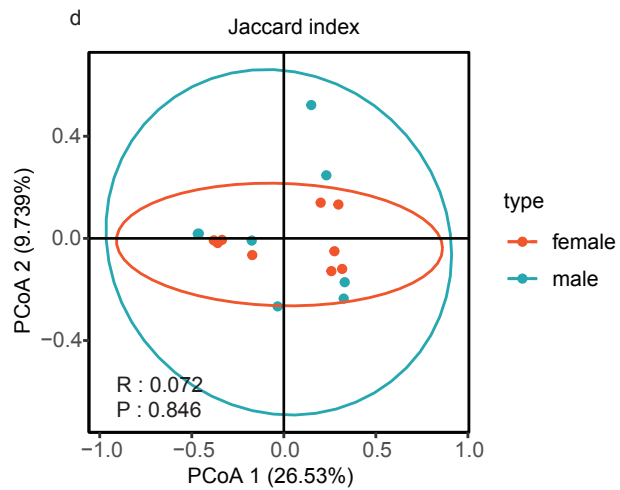

Supplement: Supplementary file 4 — Additional file 4: Supplemental Figure 4. Alpha and beta diversity of healthy ocular surface microbiota in male and female subjects. Shannon (a) and inverse Simpson indices (b) were used to estimate the level of diversity of the microbiota of the male and female groups. PCoA plots of Bray-Curtis (c) and Jaccard (d) distance matrices between the male and female groups. [file 40662_2021_228_MOESM4_ESM.pdf]

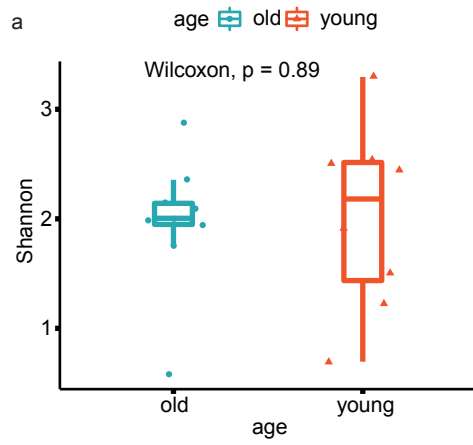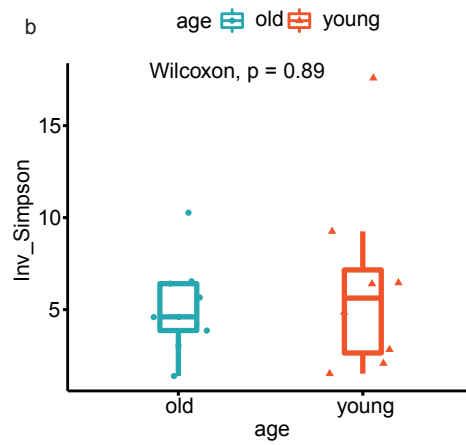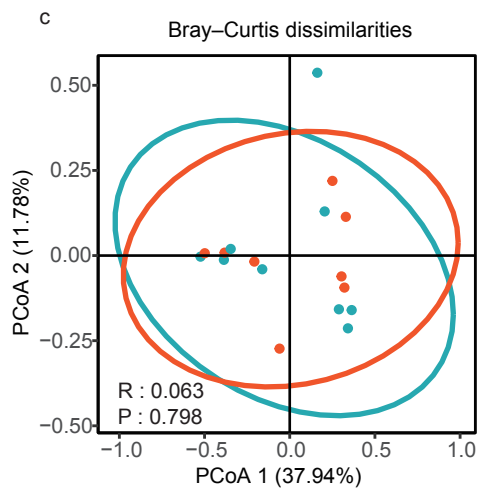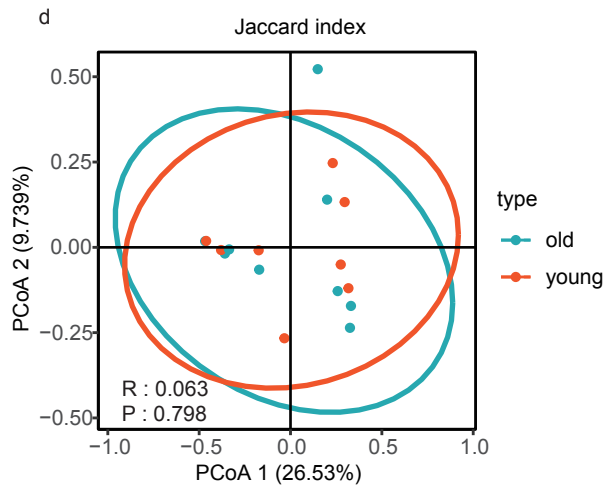

Supplement: Supplementary file 5 — Additional file 5: Supplemental Figure 5. Alpha and beta diversity of healthy ocular surface microbiota in young and old subjects. Shannon (a) and inverse Simpson indices (b) were used to estimate the level of diversity of the microbiota of the young and old groups. PCoA plots of Bray-Curtis (c) and Jaccard (d) distance matrices between the male and female groups. [file 40662_2021_228_MOESM5_ESM.pdf]
